# Monodisperse Pt-Co/GO anodes with varying Pt: Co ratios as highly active and stable electrocatalysts for methanol electrooxidation reaction

**DOI:** 10.1038/s41598-020-63247-6

**Published:** 2020-04-09

**Authors:** Hakan Burhan, Hasan Ay, Esra Kuyuldar, Fatih Sen

**Affiliations:** Sen Research Group, Biochemistry Department, Faculty of Arts and Science, Dumlupınar University, Evliya Çelebi Campus, 43100 Kütahya, Turkey

**Keywords:** Catalysis, Catalyst synthesis

## Abstract

The intense demand for alternative energy has led to efforts to find highly efficient and stable electrocatalysts for the methanol oxidation reaction. For this purpose, herein, graphene oxide-based platinum-cobalt nanoparticles (Pt_100−x_Co_x_@GO NPs) were synthesized in different ratios and the synthesized nanoparticles were used directly as an efficient electrocatalyst for methanol oxidation reaction (MOR). The characterizations for the determination of particle size and surface composition of nanoparticles were performed by transmission electron microscopy (TEM), X-ray diffraction (XRD) and X-ray photoelectron spectroscopy (XPS). The structure of the catalysts was detected as face-centered cubic and the dispersion of them on graphene oxide was homogenous (distributed narrowly (4.01 ± 0.51 nm)). Cyclic voltammetry (CV) and chronoamperometry (CA) was utilized for testing electrocatalytic activities of all prepared NPs for the methanol oxidation reaction. It was detected that the newly produced NPs were more active and stable than commercially existing Pt(0)/Co nanomaterial in methanol electro-oxidation in acidic media.

## Introduction

The researches for environmentally friendly and reusable power have been enhancing due to the gradually increasing pollution issues of the earth. Fuel cells, which are one of the alternative energy sources, are electrochemical cells that convert chemical energy directly into electrical energy with high conversion efficiency and low environmental pollution. In recent years, several studies have been conducted directly on alcohol fuel cells^1–5^. Direct alcohol fuel cells (DAFCs) are used in portable electronic devices because they are more ergonomic as energy sources. DAFCs have become popular among fuel cells because of their easy supply of alcohol derivatives, high energy densities, and relatively easy storage and transport processes. Among the direct alcohol fuel cells, direct methanol fuel cells (DMFCs) are among the most widely studied alcohol fuel types. Because methanol has a structurally simple, easily available and promising electrochemical activity. Due to these properties, many studies have been carried out on electrooxidation^6–8^. Besides, methanol has many advantageous over pure hydrogen as fuel, such as transportability, storage, cost-effectiveness and high theoretical energy density^9–11^. As in all fuel cells, the reaction mechanisms of alcohol with a catalyst that affect the efficiency of these mechanisms are one of the most important parameters in alcohol fuel cells. So far, many studies have been carried out on these catalysts^12–14^. Some of the main problems in the application of DMFCs can be listed as follows: Poisoning caused by adsorption of CO and similar intermediates formed during dehydrogenation of the methanol by catalysts (eg platinum and platinum-based catalysts). This poisoning reduces the efficiency of methanol oxidation kinetics by inhibiting the operation of active sites of catalysts. Another problem is that the platinum catalysts used are very expensive to be commercially available, as the fuel cells show superior performance in the anode section. In order to make it commercially viable, platinum was modified with different metals and more economical alloys or mixtures^15–26^. The catalytic activity and stability of the present electrocatalysts are not efficient enough to directly commercialize methanol fuel cells and make them widely available^7,27,28^. Within these catalysts, Pt+second metal bimetallic nanoparticles bonded onto the graphene oxide support were considered to be one of the most attractive electrocatalysts for the methanol electro-oxidation reaction as a direct anodic reaction of methanol fuel cells^29,30^. Therefore, various studies have been conducted to investigate bimetallic catalysts such as PtSn^31,32^, PtPb^33^, PtNi^34^, PtAu^35,36^, PtMo^37^. Catalytic performance and utilization efficiency may vary greatly depending on the performance of the platinum-based electrocatalyst in the fuel cell depending upon the type of support and metals^38–40^. Supporting and/or stabilizing agents are also very important materials in catalytic systems. They result in increasing dispersity, electron transfer, long-term stability and transport of materials in fuel cell electrodes. Nowadays, many scientists have been much interested in graphene and graphene-based supports^41–43^. In this context, various studies have been made by using Platinum-based NPs with graphene due to the very good electrical conduction and good performance of graphene^44–47^. Because graphene oxide (GO) and graphene-based materials are highly dispersible in H_2_O and a few organic solvents^48–53^. Over the years many PtCo catalysts with various ratios and morphology has been reported for catalyzing the electrooxidation of methanol^54–56^. For this purpose, in our laboratory, many studies have been carried out in order to increase the efficiency of the catalyst with the help of the addition of second metal and using of different supporting agent^53,57^. In this context, herein, GO-supported platinum-cobalt NPs were synthesized in various ratios by using the double solvent reduction method in this work. All prepared catalysts have been characterized by XRD, XPS, TEM, HR-TEM etc. in order to reveal the morphology and structure of the prepared catalysts. Further, they have been tested for their electrocatalytic efficiency towards methanol oxidation reactions.

## Experimental

The chemicals, instruments and the experimental details are given in supporting information. Graphene powder from graphite powder (GO) was synthesized using the Hummer’s method^58,59^. All prepared Pt_100−x_Co_x_@GO NPs were synthesized by a double solvent reduction method. In shortly, for the preparation of NPs with a various atomic ratio of Pt and Co (1:0, 1:1, 1:3, 3:1 ratio), the calculated amount of PtCl_4_ and CoCl_2_ as precursor materials were dissolved in tetrahydrofuran to be able to prepare the NPs and then. The prepared GO was added to the medium as stabilizing and supporting agents with a 1:1 ratio of platinum-cobalt nanoparticles. Ethanol and super hydride (Li(C_2_H_5_)_3_BH) were added up to the complete reduction of Pt and Co metals. The formation of Pt_100−x_Co_x_ NPs is understood by the observation of brown-black color in solution. Finally, the resulting solid Pt_100−x_Co_x_ NPs were dried under a vacuum.

## Results and Discussion

**Figure 1:**
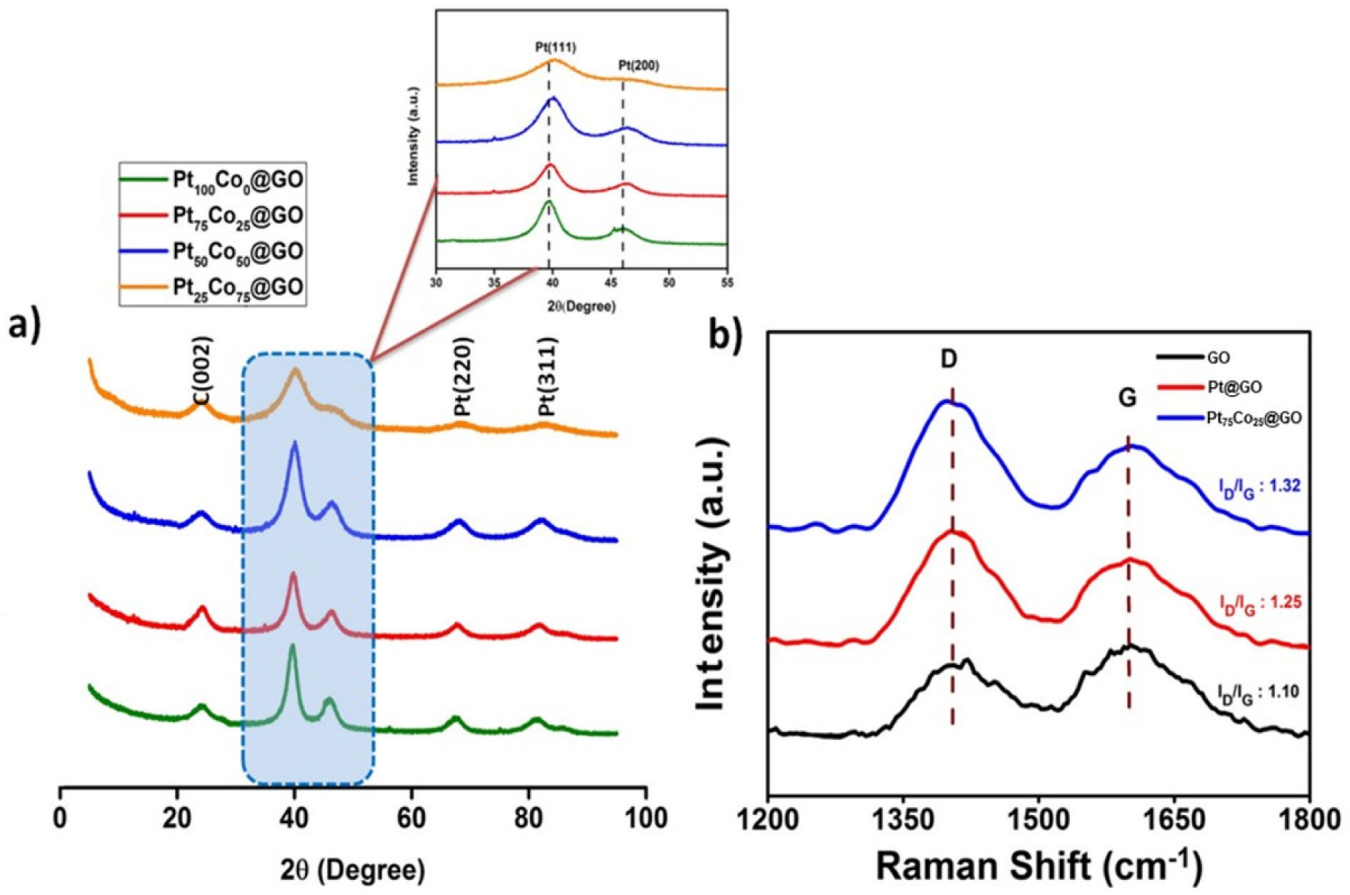
X-ray diffraction (XRD) patterns for all prepared Pt_100−x_Co_x_@GO catalysts (**a**), Raman spectra of GO, Pt@GO, and Pt_75_Co_25_@GO as a model catalyst (**b**).

All prepared catalysts were characterized by XRD, XPS, TEM, and RAMAN spectroscopy methods. XRD (X-ray diffraction) was used to determine the size of the crystallites and the crystalline structure of the prepared catalysts. In all XRD patterns, Pt (111), Pt (200), Pt (220) and Pt (311) diffraction peaks were clearly shown as an indication of the faced-centered cubic (fcc) structure of the prepared NPs. Besides, the peak at 26^o^ corresponds to C (002) plane which indicates the existence of GO. As shown in Fig. 1a, there are four diffraction peaks at values of 40.1^o^, 46.3^o^, 67.4^o^ and 81.4^o^, which are related to (111), (200), (220) and (311), respectively (Fig. 1a) (JCPDS 87–0646). Specifically, the Pt (111) peak shifts from 39.9 ^o^ (pure Pt) to 40.13 ^o^, 40.21 ^o^, and 40.30 ^o^, for Pt_75_Co_25_@GO, Pt_50_Co_50_@GO, and Pt_25_Co_75_@GO, respectively. This case indicates the alloy formation in all prepared bimetallic nanoparticles.

The characteristic plane of Pt (111) shows the crystalline structure of the nanoparticle and a shift of 2θ degree occurs with increasing cobalt concentration. This can be explained by the formation of cage shrinkage due to the integration of cobalt which is a smaller atom than Pt. The average crystallite size was calculated for Pt_100−x_Co_x_@GO using Scherrer formula.

$$d(\AA )=\frac{k\lambda }{\beta cos\theta }$$

where;

k = a coefficient (0.9)

λ = the wavelength of X-ray used (1.54056 Ǻ)

β = the full-width half-maximum of respective diffraction peak (rad)

θ = the angle at the position of peak maximum (rad)

**Figure 2:**
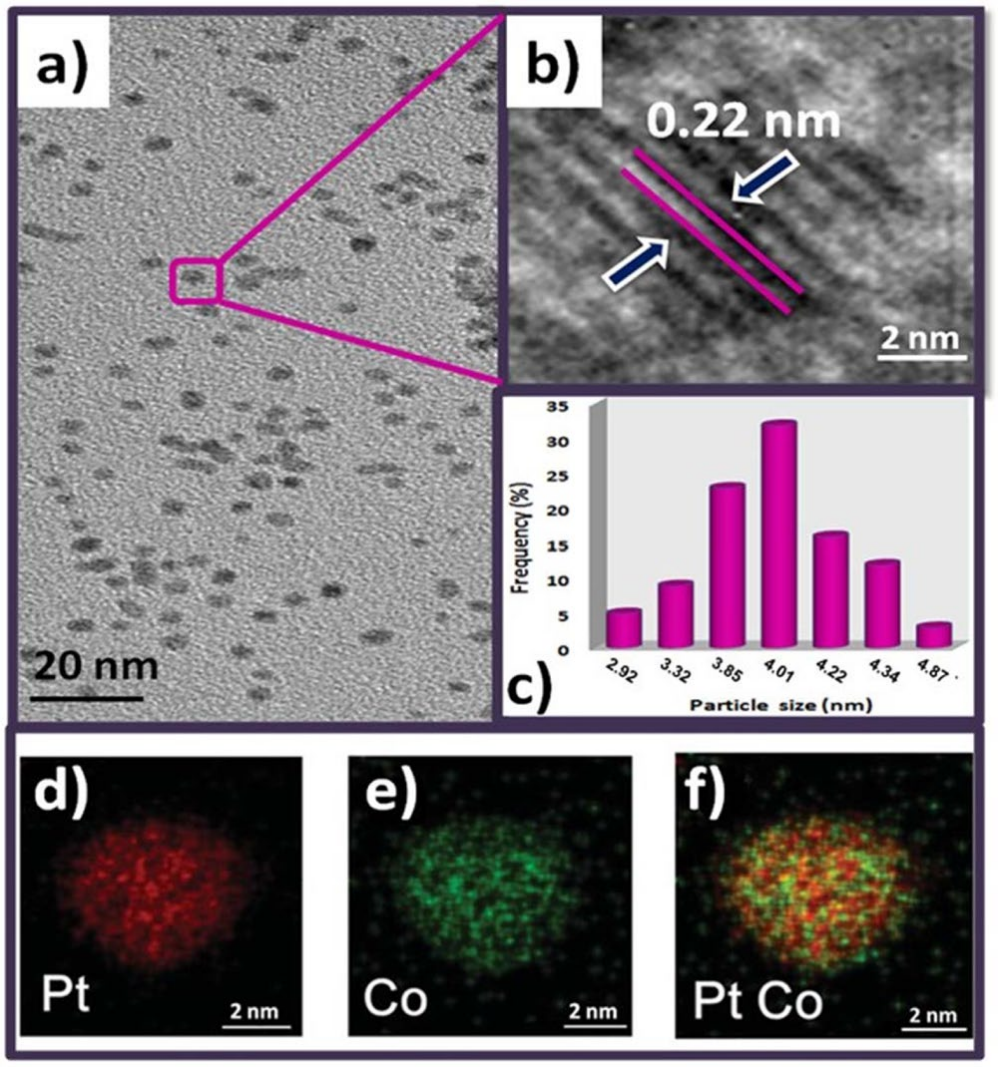
TEM (**a**) and HR-TEM (**b**) images of Pt_75_Co_25_@GO NPs with particle size histogram (**c**) EELS elemental mapping of Pt_100_Co_0_, Pt_0_Co_100_, and Pt_75_Co_25_ nanocomposites, respectively (**d–f**).

A decrease in the lattice parameter is observed as a result of the increase in Co concentration in the PtCo nanocatalyst (Table 1). This is considered to be the case that Co fills the internal spaces between Pt atoms. Raman spectroscopy was also used to visualize the changes after PtCo addition on the GO surface. Figure 1b shows the values of the D and G bands related to GO, Pt@GO and PtCo@GO. As shown in this figure, here, the G band shows the E_2g_ construction mode of the carbon atoms bound to sp^2^, while the D band shows the A_1g_ breathing mode of an irregular graphite structure. The ratios of D-and G-band (I_D_/I_G_) intensities for GO, Pt@GO, and Pt_75_Co_25_@GO were 1.10, 1.25, and 1.32, respectively. The increasing ratio of D/G band means the functionalization and/or increasing irregularity of the graphene oxide surface after the addition of PtCo NPs. The size, composition, and morphology of Pt_75_Co_25_@GO NPs are shown in Fig. 2 as a model catalyst. The morphology of the prepared catalyst is also shown in Fig. 2b with a high-resolution electron micrograph (HRTEM). From the HRTEM image, it can be said that the particles are generally spherical and do not agglomerate in the synthesized catalyst. Furthermore, the atomic lattice fringes are seen by the HRTEM image of the monodisperse Pt_75_Co_25_ NPs as shown in Fig. 2b. As a result of these fringes, a Pt (111) plane was observed on the prepared catalyst in a range of 0.22 nm; which is similar to a nominal Pt (111) range of 0.23 nm. In addition, the mean particle size of Pt_75_Co_25_@GO NPs was found to be 4.01 ± 0.51 nm (Fig. 2c) which is in good agreement with XRD results. Further, TEM-EELS mapping of Pt_75_Co_25_@GO NPs was also performed in order to see the structure of the catalyst and it’s seen that Pt and Co co-exist together which also confirms the alloy formation of prepared catalyst. In addition, the formation of an alloy composition with uniformly distributed platinum and cobalt throughout the entire nanoparticle was shown in this figure.

**Table 1:** The comparison of particle size obtained from (a) XRD, (b) TEM.

	a (nm)	b (nm)
**Pt**_**100**_**Co**_**0**_**@GO**	∼4.45	∼4.30
**Pt**_**75**_**Co**_**25**_**@GO**	∼3.87	∼4.01
**Pt**_**50**_**Co**_**50**_**@GO**	∼3.66	∼3.52
**Pt**_**25**_**Co**_**75**_**@GO**	∼3.48	∼3.21

**Figure 3:**
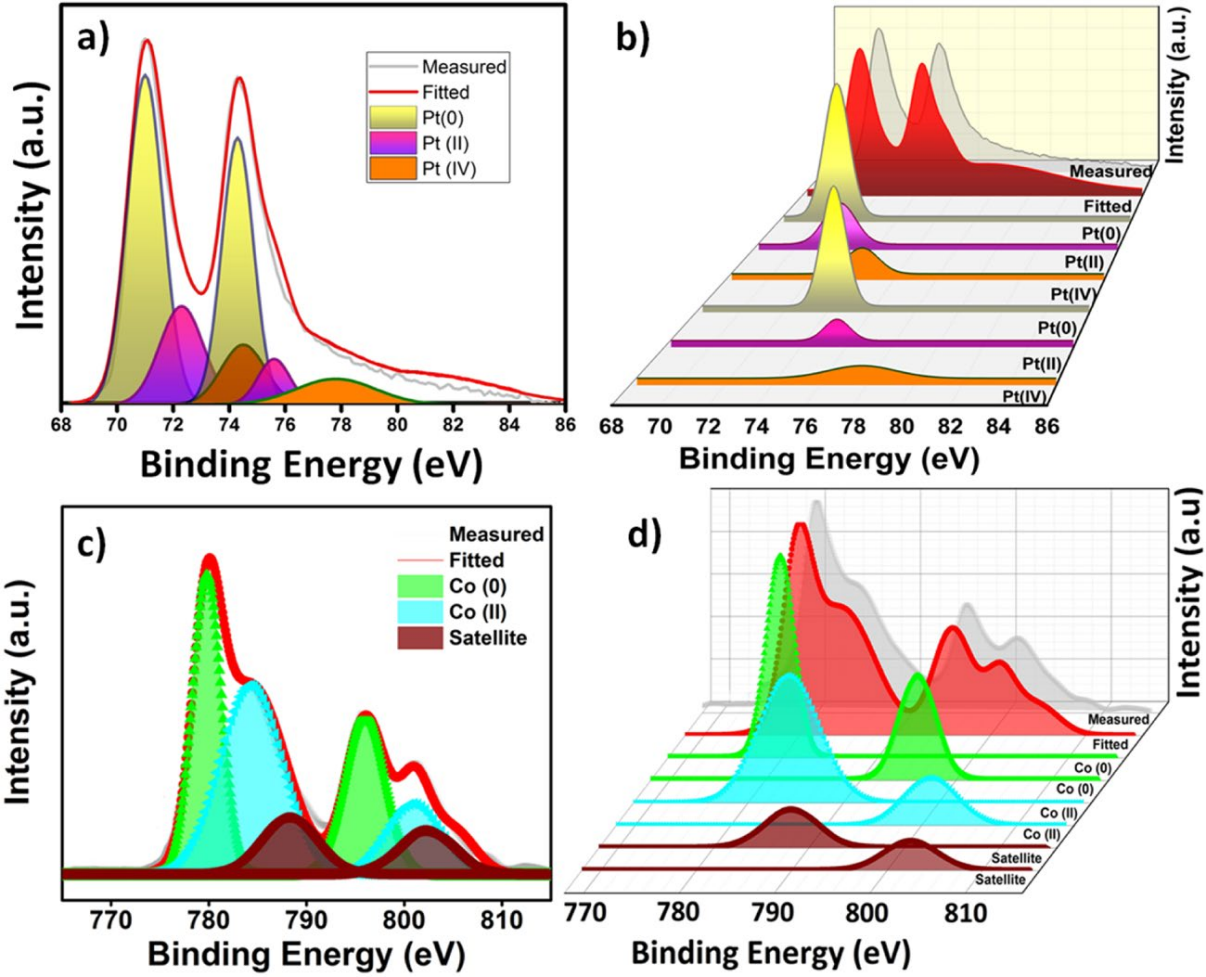
The Pt 4 f (**a,b**) and Co 2p (**c,d**) XPS spectra of Pt_75_Co_25_@GO NPs.

The surface composition and chemical oxidation states of Pt and Co in monodisperse Pt_100−x_Co_x_@GO NPs were investigated using X-ray photoelectron spectroscopy (XPS). As a result of this, the Pt 4 f and Co 2p regions of the spectrum were evaluated by the Gaussian-Lorentzian method and the relative density of the species was estimated by calculating the integral of each peak after Shirley background subtraction. The correct binding energies (± 0.3 eV) in the shaped background XPS spectrum were determined with reference to the C1s peak at 284.5 eV (Fig. S1). As shown in Fig. 3, XPS spectra show that the surface Pt and Co are found to be mostly metallic and a small amount of oxides (Fig. 3a,b). Though platinum is mainly metallic form in Pt_100−x_Co_x_@GO, from the images, the presence of PtO and PtO_2_, indicative of the oxidation of surface was understood from the existence of 2+ and 4+ species. Table S1 represents BEs of the 4f_7/2_ data for Pt_100−x_Co_x_@GO and Pt@GO and their comparative densities. It is illustrated in Table S1 that the highest amount of platinum (0) is shown in Pt_75_Co_25_@GO compared to all other prepared ones. From Table S1 and Fig. 3a,b, binding energy (for 4f_7/2_ peak) of platinum cobalt/graphene-oxide nanomaterials is 0.1–0.2 eV higher comparing to bulk platinum ones. The cause of positive change is the interaction between the final state of relaxation and platinum/cobalt-graphene-oxide. Table S1 also shows the relative intensities of metallic species in all prepared catalysts and the higher platinum (0) content (83.1%) was shown in Pt_75_Co_25_@GO compared to the other prepared Pt_100−x_Co_x_@GO. When Co 2p peaks are examined, it’s seen that cobalt is in mostly zero oxidation state at about 780 eV and in a small amount of oxidized species at about 786 eV.

**Figure 4:**
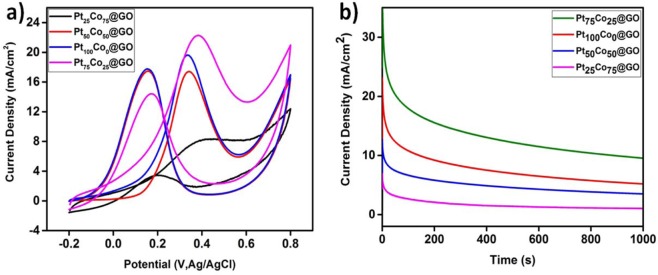
CV reaction profiles during methanol electro-oxidation of the Pt_100−x_Co_x_@GO catalysts performed in a 0.5 M CH_3_OH + 0.5 M H_2_SO_4_ solution saturated with N_2_ at 25 °C at a scan rate of 50 mV/s (**a**), CA reaction profiles during methanol electro-oxidation of the Pt_100−x_Co_x_@GO catalysts in an 0.5 M CH_3_OH + 0.5 M H_2_SO_4_ solution saturated with N_2_ at 25 °C (**b**) (0.4 V). cm^2^.

After full characterization of prepared catalysts, the electrocatalytic performance of Pt_100−x_Co_x_@GO was employed towards methanol oxidation reaction. For this purpose, the prepared electrodes with the help of nanomaterials were dipped into 0.5 M sulfuric acid in order to prepare electrocatalyst for the measurements. To obtain consistent results in cyclic voltammetry (CV), electrodes were processed among −0.2 and 0.8 V at 50 mV s^−1^. The cyclic voltammograms of all prepared Pt_25_Co_75_@GO, Pt_50_Co_50_@GO, Pt_100_Co_0_@GO and Pt_75_Co_25_@GO electrodes are illustrated in Fig. 4a. A strong oxidation peak is observed in all prepared catalysts. It is also seen from Fig. 4a that the methanol oxidation peak is situated at about 0.38 V for Pt_75_Co_25_@GO. As shown in Fig. 4a, the best catalytic performance was seen in Pt_75_Co_25_@GO electrodes which have 1.27, 1.44, 1.54 and 2.94 higher performance than Pt_100_Co_0_@GO, Pt_50_Co_50_@GO, PtRu (20%) E-TEK, and Pt_25_Co_75_@GO catalysts, respectively. The prepared best catalyst have also been compared with PtRu (ETEK), PtCo@C, PtCo@GC and it was seen that Pt_75_Co_25_@GO showed better catalytic performance compared to the others as shown in Fig. S2 and S3.

In order to explain the higher performance of Pt_75_Co_25_@GO electrodes, electrochemical surface area (ECSA), chemical surface area (CSA) and metal utility % (ECSA/CSA) were calculated as shown in Table S2. In this table, the comparison of crystalline particle size, ECSA, CSA and metal utilization (%) for the prepared catalysts are given in detail^60^. These data show that Pt_75_Co_25_@GO NPs have the highest metal utility (89.38%) compared to the other prepared ones. This case explains very well the higher performance of Pt_75_Co_25_@GO NPs than the other prepared ones. In addition, higher methanol oxidation reaction performance of Pt_75_Co_25_@GO NPs can be explained by several ways correlating with XPS, TEM, etc. X-ray photoelectron spectroscopy data in Table S1 reveals that platinum is in the more metallic state in Pt_75_Co_25_@GO NPs (83.1%) than the other prepared ones and consequently causes a more effective activation of adsorbed CH_3_OH. The higher performance of Pt_100−x_Co_x_@GO is achieved when the more metallic state of platinum is observed in the catalyst because of mixing with cobalt. Besides, the great NPs distribution on graphene oxide leads to an extensive increase in the catalytic performance of Pt_100−x_Co_x_@GO and is also the demonstration of the positive impact of utilizing graphene oxide. When all prepared catalysts were compared, Pt_75_Co_25_@GO NPs exhibited higher performance catalytically in the methanol oxidation reaction, that’s why the examination to reveal long-time stability was performed by using 0.5 M methanol and 0.5 M sulfuric acid mixture by chronoamperometry (CA) as shown in Fig. 4b. It can be concluded from the figure (Fig. 4b) that for all catalysts, the peak currents decrease during the time. However, after 3600 seconds, Pt_75_Co_25_@GO NPs have still higher catalytic activity and stability compared to the other prepared Pt_100−x_Co_x_@GO ones. Catalytic lifetime measurements of Pt_75_Co_25_@GO NPs (the best catalyst in prepared ones) are performed in a nitrogen saturated solution of 0.5 M H_2_SO_4_ containing 0.5 M CH_3_OH at a scan rate of 50 mV s^−1^ at a 1^st^ and 1000^th^ cycle (vs. Ag/AgCl). As shown in Fig. S4, when it’s compared to the current between the 1^st^ and 1000^th^ cycle, it can be said that there is only a 12.48% decrease of the initial performance of Pt_75_Co_25_@GO NPs which shows the high stability and durability of the current catalyst. It is important to investigate the high efficiency and stability of Pt-based electrocatalysts. However, it has been found that small Pt nanoparticles can easily be separated from carbon supports and their stability is discussed^21^. Stability problems in PtRu-based catalysts which are considered active catalysts for MOR activity prevent commercial use^61^. Therefore, studies are carried out to protect the catalytic stability at variable molar concentrations and the stability problem is avoided^62^. Similarly, the stability of the Pt_75_Co_25_@GO NPs, in this study is much better than catalysts formed with other molar concentrations. The Pt_75_Co_25_@GO NPs displayed good reactivity in the potential (−0.2 V to 0.8 V) with various scan rates from 50 to 250 mV/s (Fig. S5). In addition, electrical conductivity was determined by performing EIS analysis of the support material (Fig. S6). The increase in the current density with the increase in the potential scan rate is attributed to the excitation signal caused during the charging of the interface capacitance by the charge transfer process. Besides, cyclic voltammetry results showed that when the scan rate was increased, the peaks didn’t change which shows the very good electrochemical reversibility and high rate performance. Furthermore, the prepared anodic material indicates high current density and capacitance depending upon its morphology and good conductivity.

## Conclusions

As a conclusion, the synthesis and characterization of graphene oxide supported platinum-cobalt nanoparticles with different ratios (Pt_100−x_Co_x_@GO NPs) were performed. The simple double solvent reduction method was employed to produce NPs as a facile method. The structure of the catalysts was detected as face-centered cubic and the dispersion of them on graphene oxide was homogenous (distributed narrowly (4.01 ± 0.51 nm)). The altered promoter served quite dispersed metal holding parts for the nucleation of NPs on the graphene oxide’s surface, allowing a monodisperse and homogeneous distribution of Pt_100−x_Co_x_@GO NPs. The synthesized nanoparticles were used directly as anode material in direct methanol fuel cells (DMFCs). Cyclic voltammetry (CV) and chronoamperometry (CA) was utilized for testing electrocatalytic activities of all prepared NPs for the methanol oxidation reaction. The best catalytic performance was seen in Pt_75_Co_25_@GO electrodes which have 1.27, 1.44, 1.54 and 2.94 higher performance than Pt_100_Co_0_@GO, Pt_50_Co_50_@GO, PtRu (20%) E-TEK, and Pt_25_Co_75_@GO catalysts, respectively. Further, Pt_75_Co_25_@GO NPs have the highest metal utility (89.38%) compared to the other prepared ones. Besides, platinum is in the more metallic state in Pt_75_Co_25_@GO NPs (83.1%) than the other prepared ones and consequently causes a more effective activation of adsorbed CH_3_OH. The higher performance of Pt_100−x_Co_x_@GO is achieved when the more metallic state of platinum is observed in the catalyst because of mixing with cobalt. Moreover, there is only a 12.48% decrease in the initial performance of Pt_75_Co_25_@GO NPs which shows the high stability and durability of the current catalyst. Besides, cyclic voltammetry results showed that when the scan rate was increased, the peaks didn’t change which shows the very good electrochemical reversibility and high rate performance. Furthermore, the prepared anodic material indicates high current density and capacitance depending upon its morphology and good conductivity. We believe that Pt_75_Co_25_@GO NPs will be strategically used in future studies to be used in methanol oxidation reactions. In the near future, these types of materials can also be used in various applications due to their superior properties.

## Supplementary information

Supplementary information.
