# Supplementary material for: Monodisperse Pt-Co/GO anodes with varying Pt: Co ratios as highly active and stable electrocatalysts for methanol electrooxidation reaction
Source: Sci Rep. 2020 Apr 9;10:6114. doi: 10.1038/s41598-020-63247-6 (PMC7145861; doi:10.1038/s41598-020-63247-6)
Supplement: Supplementary file 1 — Supplementary information. [file 41598_2020_63247_MOESM1_ESM.docx]

**SUPPORTING INFORMATION**

**Monodisperse Pt-Co/GO anodes with varying Pt:Co ratios as highly active and stable electrocatalysts for methanol electrooxidation reaction**

Hakan Burhan, Hasan Ay, Esra Kuyuldar, Fatih Sen*

Sen Research Group, Biochemistry Department, Faculty of Arts and Science, Dumlupınar University, Evliya Çelebi Campus, 43100 Kütahya, Turkey.

E-mail: [fatih.sen@dpu.edu.tr](mailto:fatih.sen@dpu.edu.tr)

**Materials and Methods**

Platinum (IV) chloride, cobalt (II) chloride (99%), Nafion® solutions (5 wt% in low aliphatic alcohols and water) and methanol were obtained from Sigma Aldrich. All chemicals supplied were used as purchased. Deionized water (DI) was purified by analytical grading using a Millipore water treatment system (18 MΩ). All the glass materials and the Teflon coated magnetic stirrers were cleaned with aqua regia. Analytical grade chemicals (Merck) were used throughout the study. FT-IR analysis was performed by using Perkin Elmer Spectrum 2 device. The morphology and structure of the synthesized samples were characterized by transmission electron microscopy (JEOL 200 kV TEM). For TEM characterization, the prepared nanomaterials were dispersed into ethanol with sonication for several minutes and poured onto a carbon-coated 400 mesh copper grid. More than 300 particles were calculated to obtain information on the overall distribution of the prepared catalyst. In X-ray diffraction measurements (XRD), a X-ray generator (Cu Ka radiation, X = 1.54056 A) and Ultima + theta-theta high-resolution goniometer with 45 kV and 40 mA were used. X-ray Photoelectron Spectroscopy (XPS) measurements were performed by using Thermo Scientific spectrometer. Kα Mg sequences (1253.6 eV, 10 mA) were used as the X-ray source. The prepared catalyst was deposited on Cu double-sided tape (3M Inc.).


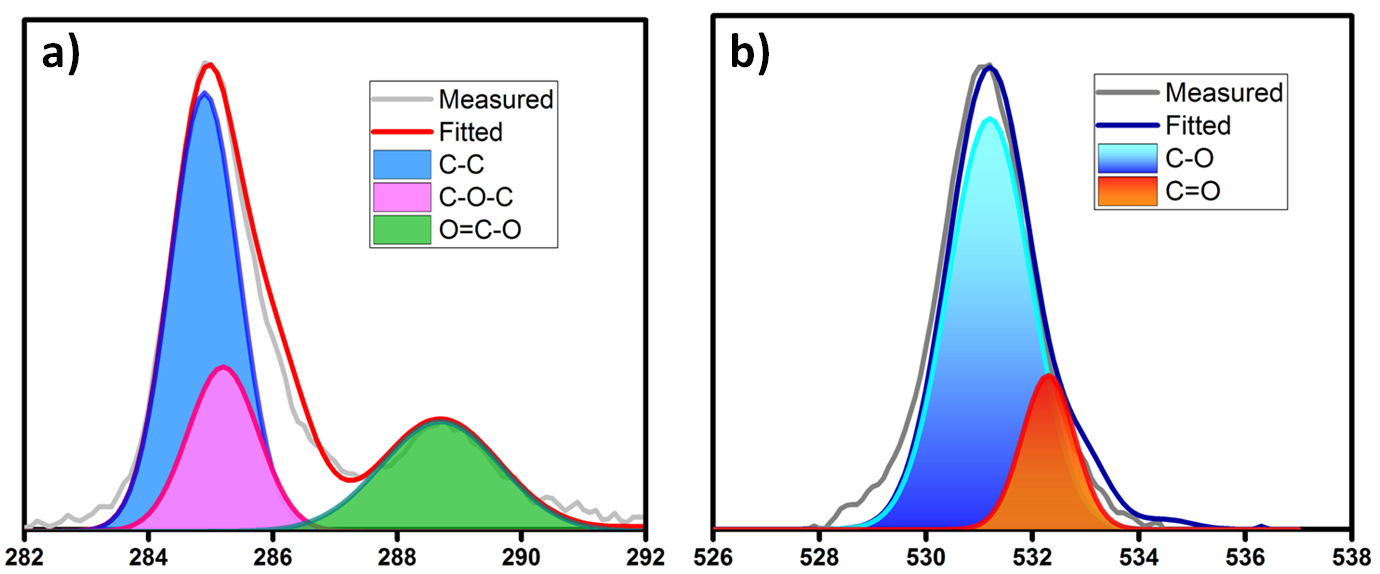


**Fig. S1.** The C1s (a) and O1s (b) XPS spectra of Pt_75_Co_25_@GO NPs.

As shown in Figure S1a , the C1s XPS spectrum of the pristine GO exhibits four different peaks centered at 284.5, 285.2 and 288.4 eV, corresponding to sp^3^ C, C = O, C-O, and O= C-O groups, respectively. Besides, in Fig. S1b, the O1s spectra of GO can be curve-fitted into two peaks, which are corresponding to C=O (carbonyl and carboxyl, 533.3 eV) and C-O (epoxy and hydroxyl, 531.8 eV).

**Table S1.** Pt 4f_7/2_ core binding energy, eV, in the prepared catalysts. The number in the parentheses is the relative intensities of the species.

|  | **Pt 4f_7/2_** | **Pt 4f_7/2_** | **Pt 4f_7/2_** |  |
| --- | --- | --- | --- | --- |
|  | **Pt(0)** | **Pt(IV)** | **Pt(II)** | **Pt(0)/Pt(IV+II)** |
| **Pt_100_Co_0_@GO** | 71.0 (80.2) | 74.4 (8.4) | 72.2 (11.4) | 4.05 |
| **Pt_75_Co_25_@GO** | 71.1 (83.1) | 74.5 (7.3) | 72.5 (9.6) | 4.92 |
| **Pt_50_Co_50_@GO** | 71.2 (78.0) | 74.6 (9.3) | 72.4 (12.7) | 3.54 |
| **Pt_25_Co_75_@GO** | 71.3 (68.1) | 74.7 (10.3) | 72.4 (21.6) | 2.13 |

MOR activities of PtCo@GC and PtRu@C catalysts were compared as shown in the figure below. The catalytic performance of the PtCo catalyst without the support material is very low. In this study, where different molar concentrations of PtCo were studied, the effect of GO supplementation on MOR activity could be demonstrated and MOR activity was superior to Pt75Co25@GO compared to PtRu@C.


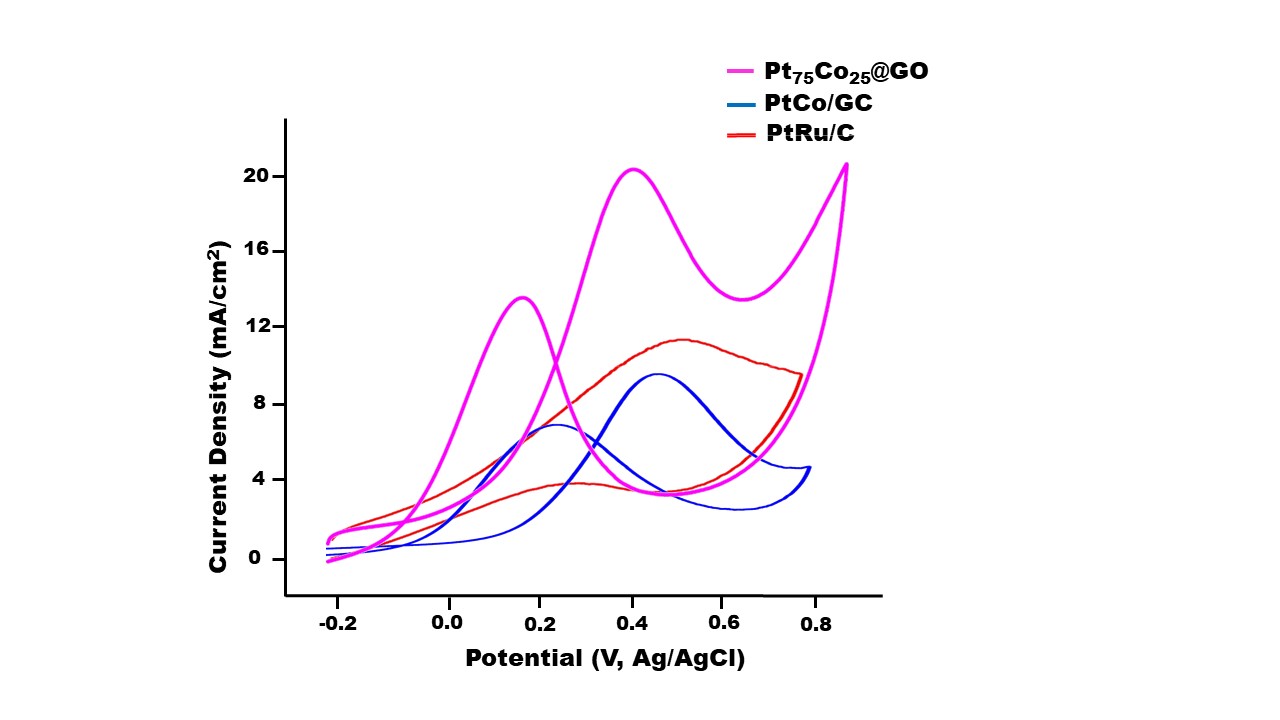


**Fig. S2.** MOR activities of PtRu@C catalysts in nitrogen saturated solution of 0.5 M H_2_SO_4_ containing 0.5 M CH_3_OH at a scan rate of 50 mV s^−1^

PtCo/GO and PtCo/C MOR activities were compared. As a result of the difference of the support material alone, it is seen that GO significantly increases the catalytic activity compared to carbon. In this study, the purpose of choosing GO as a support material is demonstrated by the difference in support material.


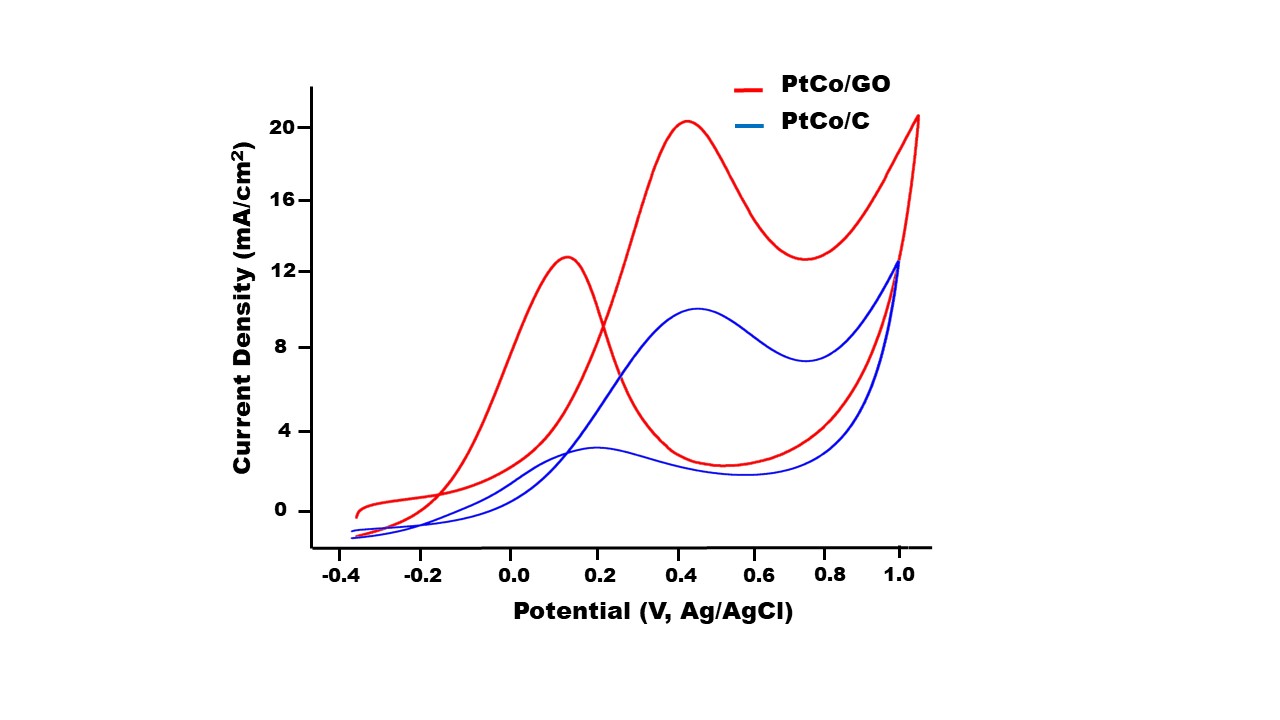


**Fig. S3.** PtCo/GO and PtCo/C MOR activities in nitrogen saturated solution of 0.5 M H_2_SO_4_ containing 0.5 M CH_3_OH at a scan rate of 50 mV s^−1^

**Table S2**. The comparison of crystalline particle size, ECSA, CSA and metal utilization for the prepared catalysts.

|  | **Particle Size (nm)** | **ECSA (m^2^/g)** | **CSA (m^2^/g)** | **Metal utility (%)** |
| --- | --- | --- | --- | --- |
| **Pt_100_Co_0_@GO** | 4.45 | 47.42 | 63.00 | 75.27 |
| **Pt_75_Co_25_@GO** | 3.87 | 64.76 | 72.45 | 89.38 |
| **Pt_50_Co_50_@GO** | 3.66 | 49.37 | 76.60 | 64.45 |
| **Pt_25_Co_75_@GO** | 3.48 | 27.59 | 80.56 | 34.25 |

The ECSA of Pt_100_Co_0_@GO, Pt_75_Co_25_@GO, Pt_50_Co_50_@GO and Pt_25_Co_75_@GO is calculated to be 47.42 m^2^/g, 64.76 m^2^/g, 49.37 m^2^/g, and 27.59 m^2^/g respectively. The CSA of Pt_100_Co_0_@GO, Pt_75_Co_25_@GO, Pt_50_Co_50_@GO and Pt_25_Co_75_@GO are calculated to be 63.00 m^2^/g, 72.45 m^2^/g, 76.60 m^2^/g, and 80.56 m^2^/g, respectively. According to the results, the catalyst Pt_75_Co_25_@GO have the largest ECSA of all the prepared catalysts due to the replacement of Pt active sites on the particle surface by Co atoms and smaller size of Pt-Co nanoparticles.


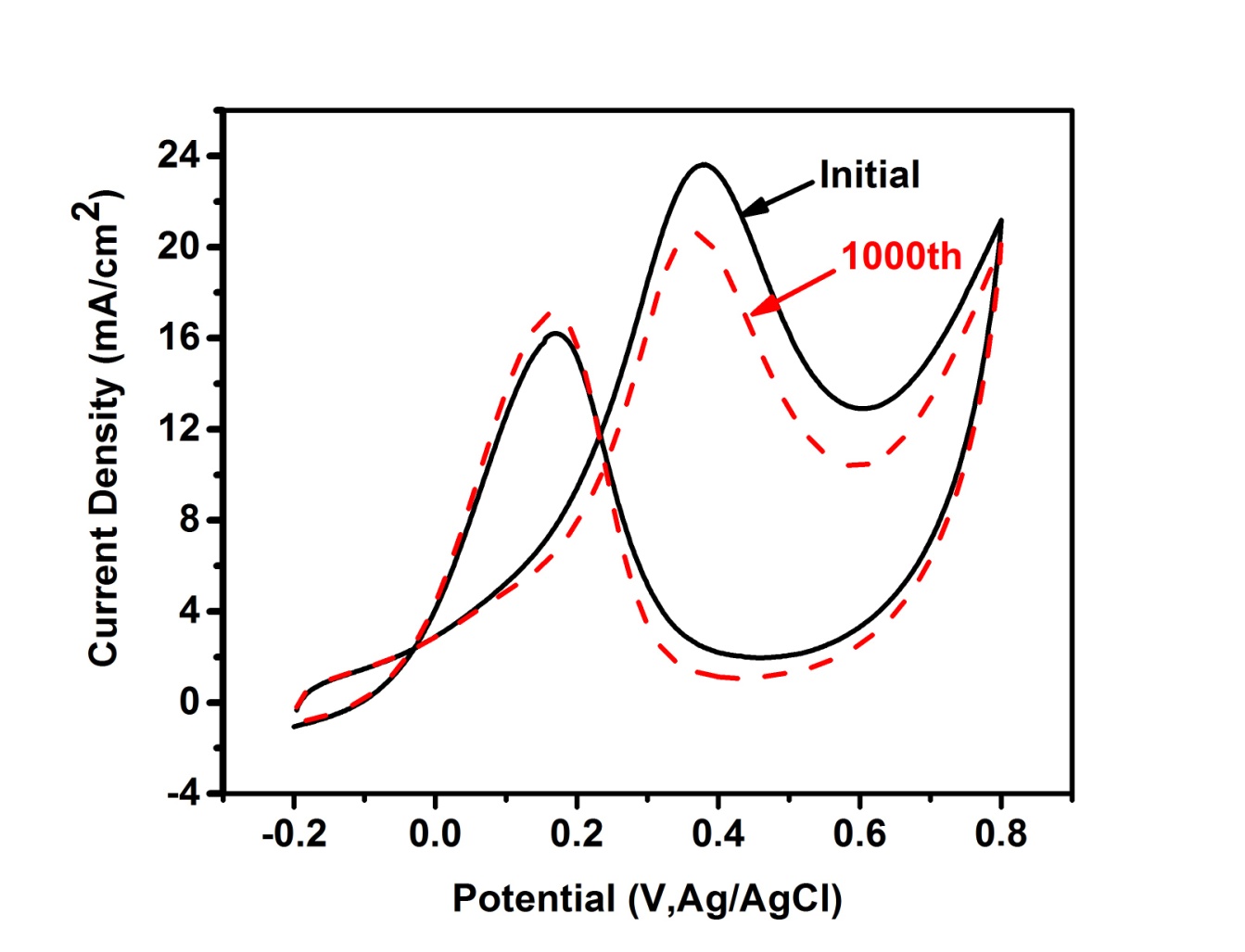


**Fig. S4.** Catalytic lifetime measurements of Pt_75_Co_25_@GO NPs (the best catalyst in prepared ones) in nitrogen saturated solution of 0.5 M H_2_SO_4_ containing 0.5 M CH_3_OH at a scan rate of 50 mV s^−1^ at a 1^st^ and 1000^th^ cycle (vs. Ag/AgCl).

The Pt_75_Co_25_@GO NPs displayed good reactivity in the potential (-0.2 mV to 0.8 V) with various scan rates from 50 to 250 mV/s. The increase in the current density with the increase in the potential scan rate is attributed to the excitation signal caused during the charging of the interface capacitance by the charge transfer process. It is further interesting to note that CV curves remain unchanged as scan rate increase thus indicating the excellent electrochemical reversibility and exceptional high rate performance. The electrode exhibits relatively high current density, corresponding to high capacitance which might be attributed to its morphology and good conductivity.


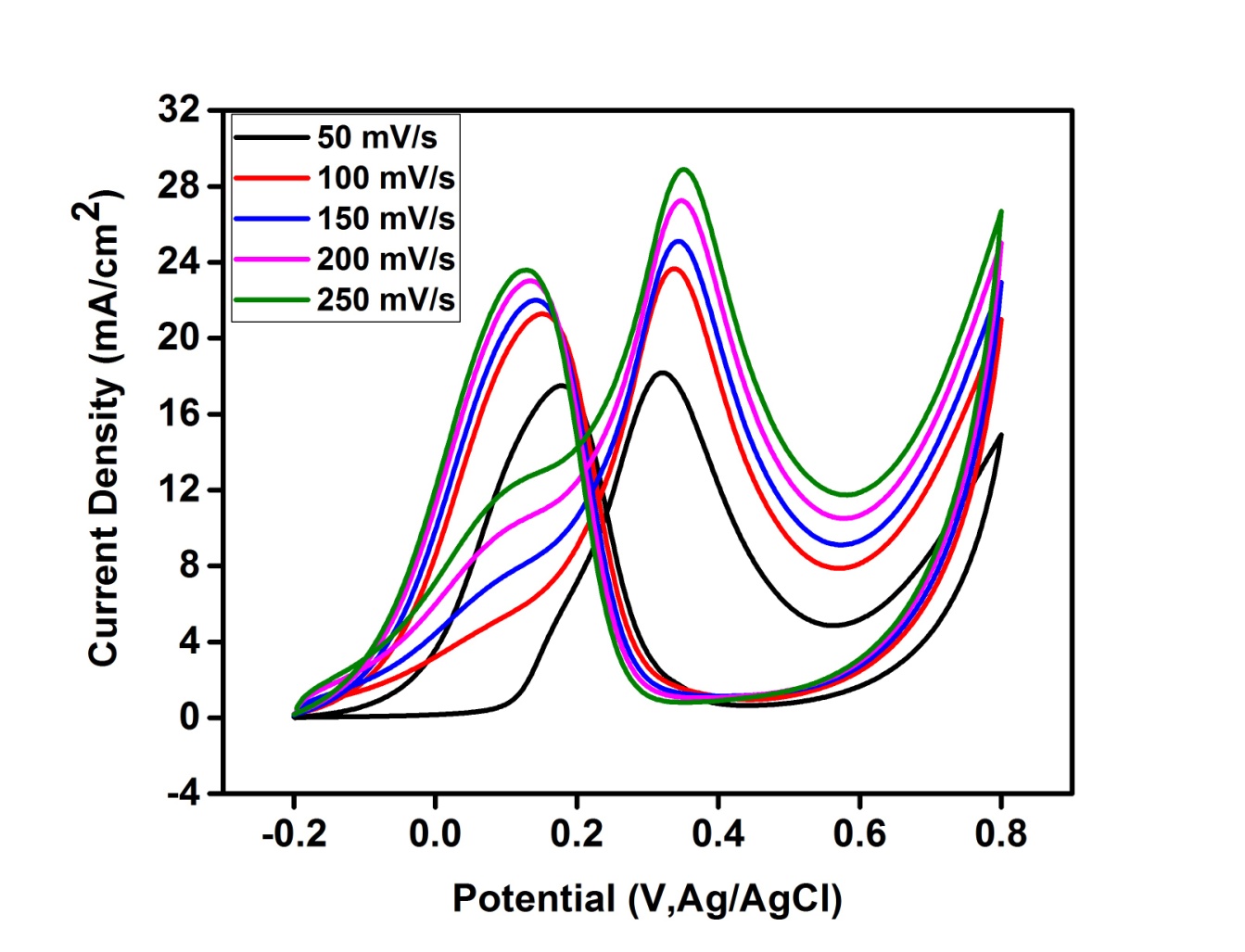


**Fig. S5.** Cyclic voltammograms of Pt_75_Co_25_@GO NPs (the best catalyst in prepared ones) in nitrogen saturated solution of 0.5 M H_2_SO_4_ containing 0.5 M CH_3_OH at different scan rates.


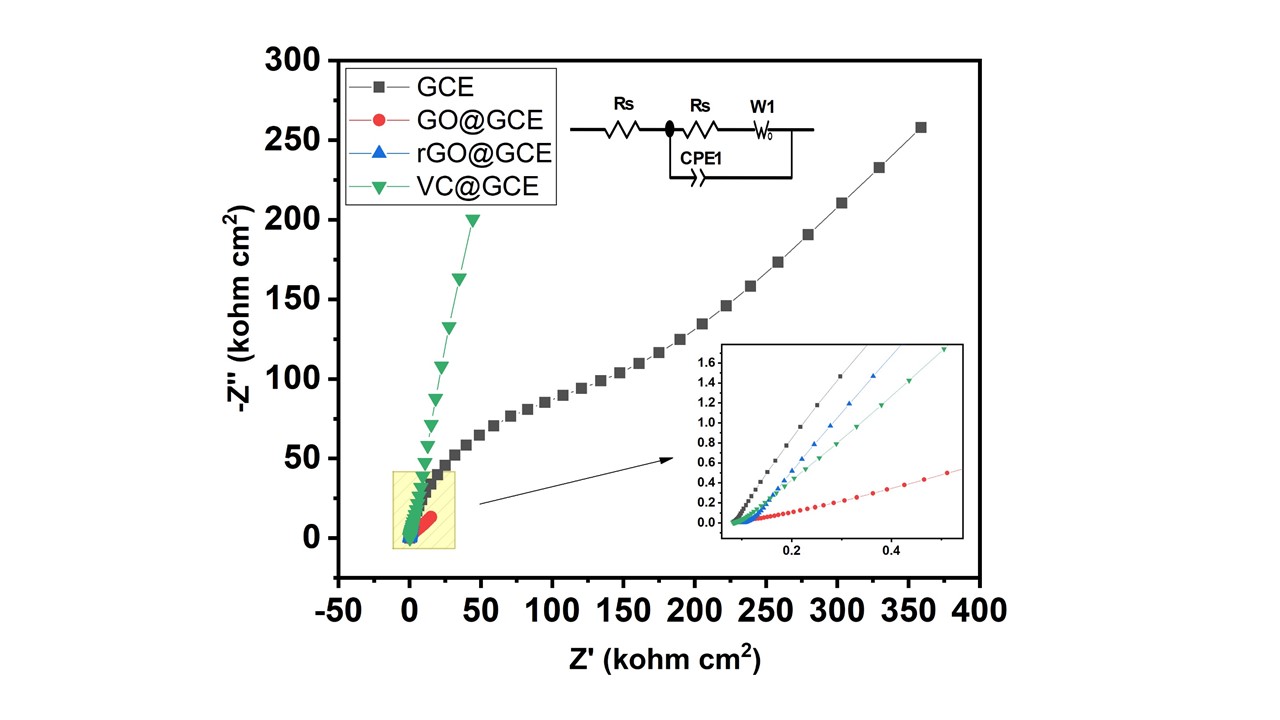


**Fig. S6.** EIS test of support materials

| **Support** | **Kohm** | **S/m** |
| --- | --- | --- |
| GCE | 127,5000 | 7.8431e-10 |
| GO@GCE | 23,47000 | 4.2608e-10 |
| rGO@GCE | 323,6000 | 3.0902e-10 |
| VC@GCE | 15,28000 | 6.5445e-10 |
